# Body perception in pregnant women: a qualitative study

**DOI:** 10.1186/s12884-023-05467-y

**Published:** 2023-03-11

**Authors:** Zahra Sohrabi, Ashraf Kazemi, Ziba Farajzadegan, Mojgan Janighorban

**Affiliations:** 1 Reproductive Health Department, Isfahan University of Medical Sciences, Isfahan, Iran; 2 Reproductive Health Department, School of Nursing and Midwifery, Isfahan University of Medical Sciences, Hezarjerib Av., Isfahan, Iran; 3 Community and Preventive Medicine Department, Medicine Faculty, Isfahan University of Medical Sciences, Isfahan, Iran

**Keywords:** Body perception, Pregnancy, Qualitative study

## Abstract

**Background:**

Dramatic body changes in pregnancy cause severe concerns among pregnant women about their appearance. Therefore, this study aimed to explore body perception in pregnant women.

**Materials and methods:**

The qualitative study, using the conventional content analysis approach, was conducted on Iranian pregnant women who were in their second or third trimester of pregnancy. Participants were selected through purposeful sampling method. In-depth and semi-structured interviews were held with 18 pregnant women aged 22 to 36 years, using open-ended questions. Sampling was performed until data saturation was reached.

**Results:**

Three main categories were extracted from 18 interviews: (1) “symbols,” with two subcategories, including ‘motherhood’ and ‘vulnerability,’ (2) “feelings toward body changes,” with five subcategories, including ‘negative feelings toward skin changes,’ ‘feeling unfit,’ ‘attention-drawing body shape,’ ‘the ridiculous body shape’ and ‘obesity,’ and (3) “attraction and beauty,” with two subcategories, including ‘sexual attraction’ and ‘facial beauty.’

**Conclusion:**

The results showed that pregnant women’s body perception could be described as maternal feelings and feminine attitudes toward changes during pregnancy compared to mental ideals of facial and body beauty. It is recommended that Iranian women’s body perception during pregnancy be evaluated using this study results and that counseling interventions be implemented for women with negative body perceptions.

**Supplementary Information:**

The online version contains supplementary material available at 10.1186/s12884-023-05467-y.

## Background

Body Image (BI) is how individuals see their body and their perception of how others view them [1] and influenced by cultural and social factors [2]. BI impacts social and interpersonal relationships by affecting self-esteem [3]. Consequently, in some individuals, despite their normal appearance, persistent preoccupation with physical appearance would cause extreme and disturbing fear of being ugly or unattractive [4–6].

BI is a multidimensional concept that involves people’s positive and negative perceptions, thoughts, behaviors, and attitudes about their body and appearance [1, 7]. BI includes perceptual and attitudinal dimensions. Perceptual dimension refers to the extent to which individuals assume that their competence is measured by their appearance. The attitudinal dimension refers to two elements: orientation and evaluation [2].

One of the processes, which causes noticeable changes, is pregnancy. Pregnancy is a period when extensive physiologic changes occur in a relatively short period of about 40 weeks, during which body changes create new aspects of body perceptions. As the body encounters new changes, the mental preoccupation with its new shape takes on new dimensions. The difference between one’s mental and ideal images forms a positive or negative body image [2], According to a systematic review, women’s body perception depends on the social construction of beauty [8], which is a mental concept.

The previous study showed that these profound physiological changes would cause first-time mothers’ bodies to distance from their mental ideals [9]. Generally, when women’s body perception during pregnancy is not accepted and leads to negative attitudes toward their bodies, their mental health might be harmed [10]. Przybyła-Basista et al.’s study showed that body acceptance during pregnancy was one of the predictors of prenatal depression [11].

Moreover, body perception distanced from mental ideals during pregnancy can lead to dissatisfaction with the body, which is associated with eating disorders [12]. The well-being of pregnant women could be harmed by eating disorders resulting from body dissatisfaction [13]. An eating disorder might be associated with insufficient weight gain during pregnancy [14, 15] and, thus, a preterm low birth weight infant [16]. In addition, there is evidence that disrupted body perception among pregnant women is associated with common pregnancy complications, such as pregnancy-related lumbopelvic pain [17].

Due to the importance of healthy behavior following changes in body perception, it is necessary to identify women’s perspectives toward better contextualizing the issue of the mental concept.

A previous study shows that while for light-skinned American women, slimness is a measure of beauty [18]. However, in Non-Western countries, particularly less developed countries such as Iran, large body sizes are accepted as a measure of beauty and are considered a symbol of better socioeconomic status [7].

Women’s distinct perspective toward pregnancy might affect their body perception in different socio-cultural conditions and there is insufficient scientific evidence for discovering different aspects of body perception during pregnancy in a traditional social context such as Iran. In this regards a qualitative approach is necessary to assess body perception during pregnancy among Iranian women. Therefore, this study was conducted to explore body perception during pregnancy.

## Method

### Study design and participants

The qualitative study was conducted on Iranian pregnant women who were in their second or third trimester of pregnancy from July 2019 to April 2020. Since truth and reality are not fixed and are formed by individuals in their specific socio-cultural context [19]; therefore, the content analysis approach was chosen. Participants were selected through purposeful sampling method. In-depth and semi-structured interviews were held with 18 pregnant women using open-ended questions. Sampling was performed until data saturation was reached.

Inclusion criteria included: ‘a singleton pregnancy without complications,’ ‘ability to speak Farsi,’ ‘no hearing problems,’ ‘no physical malformations,’ and ‘no mental or cognitive problems,’ based on the recorded information in participants’ medical files. The Ethics Committee of the Isfahan University of Medical Sciences approved this study.

### Sampling

In order to achieve maximum diversity, samples were selected using the purposive sampling method. In this type of sampling, the goal is to select individuals with a rich source of information to help the researcher gain a better understanding [20]. Moreover, to understand various aspects of the research, selecting the participants with the maximum diversity in terms of characteristics and position is essential. Therefore, to investigate the participants’ perception of their bodies, the samples were selected from a wide variety of individuals regarding characteristics (age, educational level, body mass index prior to pregnancy, employment status, and socio-economic context). Eighteen pregnant women referred to the health centers for prenatal care were selected as the study participants.

### Data collection

Data were collected through in-depth interviews, observations, and memo-writing and continued until data saturation. One researcher conducted sampling and interviews under the supervision of a reproductive health specialist. She identified the potential subjects and evaluated their inclusion criteria. The eligible pregnant women were invited to participate in the study, and the necessary explanations were provided. The time and place of the interview were determined considering the participants’ opinions.

Before performing in-depth interviews, participants’ demographic characteristics (age, educational level, employment status, gestational age, and monthly income) were recorded. Moreover, the body mass index before pregnancy was recorded based on the information from their medical file. Before the interviews, the study objective and method of implementing the interviews were explained to the participants. Besides, the researcher ensured the confidentiality of participants’ information and their volunteer participation, which had no impact on their care. At all stages of the study, participants’ values and decisions were respected. They were also allowed to withdraw from the study at any stage.

After obtaining informed consent from the participants, face-to-face, semi-structured, in-depth interviews were conducted in a private room at health centers or participants’ houses or workplaces based on their preferences. Interviews started with the open questions: “Describe your pregnant body.” and “Explain your feelings about your body.” The interview questions were developed using the opinions of two psychologists experienced in the body image field and consulting the research team.

Based on the provided information by the participants and in order to clarify the study objective, the following questions were asked: “Please explain more.” “Give an example,” and “Where did this feeling originate from?” The duration of the interviews was determined based on participants’ interest, from 60 to 90 min.

In order to ensure the trustworthiness of the data, an effort was made to employ methods to maintain credibility, dependability, transferability, and conformability. Therefore, by spending a sufficient amount of time collecting the data and repeatedly reviewing them, using maximum diversity in sampling (selecting participants with diverse demographic characteristics), providing participants with feedback on interviews, and obtaining their confirmation on the categories extracted from the interviews, the credibility of the data was improved. In order to establish the stability of the data, the data dependence method was used. In other words, the interview transcriptions were provided to the qualitative research experts to confirm the relevance of the content and results.

### Data analysis

The interviewer and supervisor analyzed the data using the conventional qualitative content analysis approach and Granheim and Lundman’s method [21]. The researchers transcribed the interviews verbatim after repeatedly listening to them. Afterward, they read the transcripts several times to gain a general idea. All interviews and observations were considered analysis units. Related words, sentences, or paragraphs were considered meaning units. Afterward, codes were compared with each other regarding their similarities and differences and were categorized under more abstract categories. A second researcher analyzed the transcripts and interviews to confirm the reliability of the findings.

Based on the observations during meetings and communications with participants, their non-verbal reactions were noticed and recorded immediately after the interviews and analyzed. All of the interviews and observations were conducted by one researcher. Three individuals cross-coded the data, and data analysis was performed simultaneously with data collection.

## Results

In this study, 18 pregnant women were interviewed. Participants’ demographic characteristics are provided in Table 1. According to the results, pregnant women’s body perception was categorized into three main categories: “symbols,” “feelings toward body changes,” and “attraction and beauty.” The category; ‘symbols’ was derived from the following subcategories: ‘symbol of motherhood’ and ‘symbol of vulnerability.’ The category of ‘feelings toward body changes’ was derived from the subcategories: ‘negative feelings toward skin changes,’ ‘feeling unfit,’ ‘attention-drawing body shape,’ ‘the ridiculous shape of the body,’ and ‘obesity.’ The category of attraction and beauty was derived from the subcategories: ‘sexual attraction’ and ‘beauty’ (Table 2).

**Table 1:** Demographic characteristics of the participants

Participant’s No.	Age	GA	BMI Kg/m^2^	Educational level	Employment status	Monthly income (Rials) ^*^
P1	26	24	24.20	Bachelor’s degree	Housewife	10–20 million
P2	32	32	20.20	Bachelor’s degree	Employed	> 30 million
P3	34	26	23.43	Bachelor’s degree	Employed	10–20 million
P4	22	22	18.07	Bachelor’s degree	Housewife	10–20 million
P5	33	28	20.30	Bachelor’s degree	Housewife	> 30 million
P6	33	36	17.64	Diploma	Housewife	10–20 million
P7	29	33	24.50	Bachelor’s degree	Housewife	10–20 million
P8	35	33	23.07	Diploma	Housewife	< 10 million
P9	35	35	25.08	Diploma	Housewife	10–20 million
P10	26	11	20.01	Diploma	Employed	10–20 million
P11	30	37	23.03	Bachelor’s degree	Employed	10–20 million
P12	28	34	23.40	Diploma	Housewife	10–20 million
P13	32	33	26.40	Bachelor’s degree	Employed	> 30 million
P14	26	37	21.42	Diploma	Housewife	< 10 million
P15	36	37	28.50	Diploma	Housewife	10–20 million
P16	22	38	22.50	Diploma	Employed	10–20 million
P17	26	28	23.20	Diploma	Employed	10–20 million
P18	24	34	29.40	Master’s degree	Employed	10–20 million

*One-dollar equivalent to 70,000 Rials

*GA* gestational age, *BMI* Body mass index

**Table 2:** The main categories, sub categories and abstracted sub-categories

Main category	Subcategory
Symbols	Symbol of motherhood
	Symbol of vulnerability
Feelings toward body changes	Negative feelings toward skin changes
	Feeling unfit
	Attention drawing body shape
	Ridiculous shape of the body
	Obesity
Attraction and beauty	Sexual attraction
	Beauty

### Symbols

This category was derived from two subcategories: (1) a symbol of motherhood and (2) a symbol of vulnerability.

#### A symbol of motherhood

Pregnant women had a maternal feeling toward the changes in their bodies, especially their growing abdomen. Most of them stated that those changes triggered their motherly feelings. A 26-year-old pregnant woman said: “*I have a good feeling toward my body because I am going to be a mother. I am deeply fond of the baby growing inside me; that is why I love the growth of my abdomen*.” (P1).

#### A symbol of vulnerability

Some pregnant women believed that the changes in their bodies during pregnancy were signs of vulnerability. A 28-year-old pregnant woman mentioned: “*My abdomen is too big. I cannot even see my feet. Walking has become too hard for me. I am always afraid of falling. I am always stressed out. Somehow, I am taking care of myself and my baby. I would rather stay at home all the time*.” (P14)

### Feelings toward body changes

This category was achieved from five subcategories: (1) negative feelings toward skin changes, (2) feeling unfit, (3) an attention-drawing body shape, (4) the ridiculous shape of the body, and (5) obesity.

#### Negative feelings toward skin changes

Pregnant women expressed irritation from changes on different parts of their skin and believed that color changes and stretch marks on their abdomen were unattractive. Moreover, color changes, stretch marks on the breasts, and darkened skin of the underarm and neck caused negative feelings. A 35-year-old pregnant woman stated: “*My underarms and sides of my breasts have become darker, and my thighs look bluish. I am worried that I cannot get rid of them. I do not want anyone to see them because they look ugly*.” (P8)

#### Feeling unfit

Pregnant women believed the changes were a factor in losing fitness and unpleasant body shape. Some women mentioned that they would compare their body shape with others and the standards for beauty in the media. The former clothes that no longer fit them would intensify their sense of being unfit. In this regard, a 29-year-old woman said: “*My abdomen has become big. I have gained weight. I look awful, and I think my appearance has become ridiculous. My back has dented, and my abdomen has stuck out. I do not want to look at myself in the mirror. I am fat and out of shape. I feel like I have gained extra fat all over my body*” (P7).

Moreover, pregnant women needed to hide the changes due to their breasts’ enlargement and sagging. A 26-year-old pregnant woman mentioned: “*In the past, everything was good, and I was fit. Now, everything looks lumpy. My breasts have become big. It makes me sad that my body shape is ruined*.” (P17)

#### An attention-drawing body shape

Women in the present study stated that their enlarged bellies and hips during pregnancy would draw others’ attention, making them uncomfortable. In this regard, a 26-year-old pregnant woman said: “*My hips have become too wide and large. They look terrible! With these hips, I look ugly and unfit. It seems that my lower parts have become loose. I always try to put on a loose T-shirt to cover my hips. I think that everybody is looking at my hips*.” (P17)

#### The ridiculous shape of the body

Some women considered their body shape ridiculous and believed those changes would look ridiculous from others’ point of view. A 36-year-old woman stated: “*My flanks have enlarged. I do not have a good image in my mind. I do not know what to say, but I think that I look like a pear with a wide bottom*.” (P15)

#### Obesity

Some women were satisfied with their weight gain and abdomen growth and considered it attractive. Others had negative feelings toward it. In this regard, a 26-year-old woman said: “*I feel that I have gained weight only in my lower limbs. My upper body looks the same. My thighs have become very fat. When I look at myself in the mirror, I can understand exactly how big and fat my hips and legs are*.” (P 10)

### Attraction and beauty

This category was derived from two subcategories: (1) sexual attraction and (2) facial beauty.

#### Sexual attraction

Some women mentioned that changes during pregnancy, especially in their breasts and genital area increased their sexual attraction. In this regard, a 33-year-old pregnant woman mentioned: “*My breasts have become larger; I am so happy about it. Because my breasts were too small before, but now they look attractive. I think my husband finds me more gorgeous now. All men like this kind of change*.” (P 5)

However, other women believed that those changes decreased their sexual attraction. In this regard, a 33-year-old pregnant woman said: “*It makes me uncomfortable that my genital area has become dark. This darkness is obvious, and I feel ashamed when my husband sees it. I feel that my sexual attraction during intercourse has decreased.*” (P 10)

#### Facial beauty

Some participants were pleased with the changes in their faces and considered themselves prettier. They believed that others likewise considered them more good-looking. In this regard, a 32-year-old woman said: “*During the first few months, I thought I became more beautiful. I thought my skin was brighter. My eyes were more distinctive, and my lips were redder.*” (P 13)

Some other women were dissatisfied with the changes, especially in their noses and lips. These women mentioned that using no cosmetics due to their possible harm to the infant made them unattractive. Moreover, attending friend gatherings and workplaces without wearing makeup increased their dissatisfaction. In this regard, a 28-year-old woman said: “*My nose has become too big and ugly. My lips have an ugly bluish look. It is not pretty at all. I prefer to have only a big abdomen during pregnancy so that others understand I am pregnant. Besides all the weight I have gained, the nose and lips are the worse.*” (P 12)

## Discussion

The present study was conducted to explore body perception in pregnant women. The present study indicated that body perception would induce a sense of motherhood under the influence of women’s feelings toward pregnancy. Along with the body perception, changes in different body parts would create a new body perception leading to negative or positive feelings.

Results of this study revealed that some changes, such as the growth of the abdomen, symbolically led to the feeling of motherhood which helped mothers adapt to changes. In the study by Clark et al., most pregnant women positively adapted to the changes during pregnancy. Participants in that study stated that the fetus’s movements and experiencing a new sensation influenced their satisfaction with the changes during pregnancy [22].

This result might indicate mothers’ bond with their growing embryos. In line with the results of the present study, Bergbom et al. report that, since changes during pregnancy are a sign of fertility, they are associated with a pleasant feeling for pregnant women [23].

In the study by Whitaker et al., some pregnant women expressed satisfaction with their growing abdomen since they considered it a sign of pregnancy progress and the growth of the fetus [24]. Nash et al. believed that pregnant women considered the growth of their abdomen as a unique pregnancy change with which other changes are associated [25].

Women’s pleasant feeling toward their body changes during pregnancy might indicate their positive attitude toward pregnancy since some reports have shown that accepting new roles following pregnancy, especially the existence of the fetus, is one of the critical factors in accepting changes during pregnancy [9].

Along with maternal feelings created by pregnancy changes, especially the growth of the abdomen, a sense of vulnerability and the need to protect the fetus were also created. Most mothers felt that the changes, especially their enlarged abdomen, would expose the fetus to various damages. This issue has not been addressed in any other study.

In addition to the symbolic motherly perception women mentioned about their pregnancy, other dual emotions expressed were placed in the ‘perceptions of body changes’ category. Women in the present study expressed negative and positive perceptions of the changes during pregnancy. Most women had a negative body perception of the changes on their skin, especially stretch marks on their bellies and legs, which created an unpleasant perception of the skin.

The review by Meireles et al. showed contradictory and inconclusive findings related to body image in pregnant women. Some studies show an improvement in body dissatisfaction among pregnant women. On the other hand, dissatisfaction with body shape and weight among pregnant women was also underlined in other studies [26].

Pregnant women’s other body perception was ‘unfitness.’ They described the changes in their bodies as disproportionate. Some participants described their breasts as too big, which did not fit their body size. Earle et al., in their study, mentioned that while most pregnant women were pleased with their enlarged breasts, others were dissatisfied due to extensive breast enlargement and physical discomfort [27]. Hodgkinson et al. reported that pregnant women perceived their body changes as transgressing the socially constructed ideal and feminine beauty. They also considered the physical manifestation of the mothering roles incongruent with their roles as a partner [8]. Another result showed that enlarged body size in different parts during pregnancy created a ridiculous body shape in women’s minds. A study has stated that women feel ashamed of their enlarged bellies and hips during pregnancy. These women were concerned that the increased size would persist after delivery. Although the increased body size following pregnancy is an expected change, some women describe it as obesity.

The concept of obesity in the present study was in line with the study by Whitaker et al. They reported that women considered weight gain during pregnancy as a factor in losing self-confidence. It was also reported that the opinions of the husband, physician, parents, and friends impacted the formation of that perspective [24].

Haruna et al., in their study, revealed that, due to healthcare providers’ emphasis on controlling weight gain during pregnancy, pregnant women mostly felt disappointed. These women believed that healthcare providers’ extensive focus on weight gain would affect their satisfaction with the body in the long term [28]. These results indicated that women would require more information to distinguish body changes during pregnancy from obesity.

Another description of the body during pregnancy was women’s opinions about sexual attraction, especially following breast enlargement. This issue was mostly observed in women who were dissatisfied with the small size of their breasts before pregnancy. These women stated that the enlargement of their breasts increased their sexual attraction. In the study by Watson et al., participants stated that their husbands found them more attractive sexually [4].

Another finding of the present study indicated that facial changes created a perception of having a prettier face in some women. Consequently, they described their faces as more good-looking. In the study by Harper and Rail, women mentioned that extra weight distributed in their abdomen, hips, and thighs was acceptable; however, if it appeared on their faces and arms, they would feel dissatisfied [29]. Some of the changes in the face, such as fattening or enlargement of the lips, might be under women’s standards for beauty; however, if they were accompanied by nose swelling and melasma, it would lead to the unattractiveness of the face. Therefore, individuals’ standards for facial beauty could affect their perception of the face. It is necessary to interpret the results of the study considering certain limitations. An unwanted pregnancy can affect women’s perception of their pregnant body, which was not considered in this study. Other study limitations were including low sample size, lack of quantitative measures, and no fidelity checks within data analysis.

## Conclusion

The study results showed that pregnancy in Iranian women is experienced through a symbolic view of body changes, feelings toward changes, and attractiveness and beauty. Subcategories of symbols included understanding maternal symbols and vulnerability. Moreover, the subcategories of feelings toward changes and perception of attractiveness and beauty included positive and negative feelings toward the physiological changes during pregnancy, which might be associated with negative judgment about the body and affect the pregnant women’s mental health. The findings of this study could be used to help develop an educational and counseling program for pregnancy.

## Electronic supplementary material

Below is the link to the electronic supplementary material.

**Supplementary material 1:** Peer review report.

## Data Availability

Data and material are available on request from the corresponding author. The peer review history is available as Supplementary file 1.

## Abbreviations

GA: Gestational age
BMI: Body mass index
