# Supplementary material for: Body perception in pregnant women: a qualitative study
Source: BMC Pregnancy Childbirth. 2023 Mar 11;23:165. doi: 10.1186/s12884-023-05467-y (PMC10007813; doi:10.1186/s12884-023-05467-y)
Supplement: Supplementary file 1 — Supplementary material 1: Peer review report. [file 12884_2023_5467_MOESM1_ESM.docx]

# **Reports v1**

Reviewer 1

The theme discussed in the article is relevant and is within the scope of BMC Nursing.

Please, see detailed comments bellow:

Abstract:

Line 29: Background is too generative.

Line 32: second / 23

Introduction:

Line 59: Background

The authors use body perception, body acceptance, and body dissatisfaction without properly defining the terms. They are not synonymous, although sometimes in the Background section, the authors tended to understand they could be used interchangeably.

Body image has 2 dimensions: perceptual and attitudinal (which also includes the behavioral, affective, and cognitive components). The authors must understand and let clear which dimension and component is going to be covered in the study.

Line 64: The results of Harrison et al research also indicated that pregnant women are concerned about returning to their bodies before pregnancy

The sentence is not coherent with Harrison et al’s findings. In addition to that, the population studied was young (18-24 years) with mean age of 19 years old. Their results should not be generalized.

Line 66: body Acceptance by pregnant women and prenatal depression

Body acceptance and prenatal depression have a negatively relationship in pregnant women. The way it is written does not show it.

Line 67: Body encounters new changes, including body perception, changes suddenly finds new aspects.

The phrase is not well formulated.

Line 70: These extreme physiological changes would cause women’s bodies to distance from their mental ideals.

Although the phrase refers to the pregnancy, the authors cite a study that included just first-time mothers.

Line 72 to 76: Body perception during pregnancy is of great importance due to direct and indirect effects on the and psychological wellbeing of the mother...

The sentence is too long and confused.

Line 83: … there is not sufficient scientific evidence for discovering different aspects of body perception during pregnancy.

There are a fair amount of publications that evaluated body image in pregnant women. Why do the authors say that there is not enough scientific evidence?

Line 85: naturalism approach...

In the abstract, it is mentioned “qualitative study, with content analysis approach”.

In general, Introduction does not establish the background of the problem studied.

Methods:

Line 88: This qualitative study is a part of a larger research study

Which study? What was the main objective and outcome?

Line 88: using content analysis approach

What is the author that explains this approach? Reference is needed.

Line 97: Samples were selected using purposeful sampling and to have maximum diversity

Since it is a qualitative study, it is not clear how and why you have done it.

Line 118: following questions were asked

Were the questions pre-established? Could you give more examples of the questions? Authors should provide more information about these questions: who did develop the questions? What were the aims of the questions?

Line 118: Questions of the next interviews were asked to complete information about the extracted categories.

Not clear what the authors meant to say.

Line 126: The transcripts and interviews were analyzed by a second researcher to provide an indication of reliability of the findings.

This information is part of data analysis.

Line 126 to 130: The transcripts and interviews were analyzed by a second researcher to provide an indication of reliability of the findings. To achieve stability of the data, data dependence method was used; meaning that the texts of the interviews were given to the experts of qualitative research to confirm the relevance of the content and the results.

There are some missing information related to the procedures of the data collection. Who did interview the participants? Was always the same person? How was the interview scheduled?

Line 140: All of the interviews and observations were conducted by one researcher

This is procedures of data collection.

Results:

Line 155: symbol of motherhood

It is missing the name of the subcategory: Symbol of Motherhood.

Discussion:

Line 233: Results of the present study showed that body perception during pregnancy are a complex concept.

The perceptual dimension of body image is related to the accuracy of the analysis of body size, shape, and weight (Gardner, Stark, Jackson, &amp; Friedman, 1999). In other words, body image perception consists of the accuracy (or inaccuracy) of judging aspects of physical appearance. To assess it, researchers have used tools that seek to assess whether or not the body image of the investigated subject is distorted, such as photos and footage (Gardner, 2011). The data collected in the present study does not represents body perception.

Line 259: Women in the present study expressed negative and positive perceptions about changes that occurred during pregnancy.

This paragraph should compared the study’s findings with previous studies. For example, review developed by Meireles et al (2015) showed contradictory and inconclusiveness findings related to body image in pregnant women. Some studies show an improvement in the matter of body dissatisfaction among pregnant women. On the other hand, dissatisfaction with body form and weight among pregnant women was also underlined in other research studies.

Line 302: A limitation of the present study was the effect of the social culture and beauty criteria in different communities

Highly recommend to review the limitations of the study.

Line 303: would reduce the generalizability of the present results for various communities.

Were the authors looking for a generalization of the results? I would discourage it. The goal of most qualitative studies is not to generalize but rather to provide a rich, contextualized understanding of some aspect of human experience through the intensive study of particular cases

Line 304: Also, low sample size, lack of quantitative measures, and no fidelity checks within data analysis are other limitations of the study.

Low sample size is a characteristic of qualitative studies. The authors said they gathered data until saturation. Then, why would a low sample size be a limitation of the study?

The study design was qualitative. Why would a lack of quantitative measure be a limitation of the study?

Conclusion

Line 306: Practical implications should be explicated.

Line 307 to 310: body perception of pregnant women could be described by comparing their bodies to their mental ideals of the face and body beauty. If this mental image is far from the formed ideals in the sociocultural context of the society, it will lead to the creation of negative body perception during pregnancy.

These sentence do not answer the aim of the study. Once again, data cannot be generalized.

References

Line 340: It is recommended to review references since just 7 were published in the past 3 years.

Recommendations for Authors

Does the Introduction provide sufficient background and include all relevant references? Must be improved.

Is the research design appropriate? Yes.

Are the methods adequately described? Must be improved.

Are the results clearly presented? Yes.

Are the conclusions supported by the results? Yes.

English language and style: Must be improved.

Top of Form

## Reviewer 2

I appreciate the opportunity to review this interesting manuscript.

I hope to be able to help in the adequacy of the text.

Some comments follow below.

Title

I suggest this version:

Body perception in pregnant women: a qualitative study

Abstract

The method needs to appear in a more organized way, I suggest you follow the sequence in which things happen in the research, here is a list of topics in the sequence that seems most appropriate to me: qualitative study. use of open-ended questions script with in-depth interview technique . with intentional sampling and closing through the saturation of information.com use of content analysis for data processing.

I believe that the number of participating women must go to the results section of the abstract and that the categories can be numbered or lettered so that the reader can better visualize what is in one category and what is in another.

Background

I believe this sentence should be placed at the end of the second paragraph.

Also, the relation between body Acceptance by pregnant women and prenatal depression has been documented [7, 8].

In the last paragraph, I believe it is necessary to write that it is necessary to know the perspective of women in order to better contextualize the issue of the mental concept that the authors bring up in the text.

Methods

Study design and participants

This subtitle needs to be revised, as it does not talk about the study design, but about the sample.

This sentence is about data collection, needs to be relocated:

Data were gathered through in-depth interviews, observations and memo-writing; data gathering continued until data saturation.

As for the number of participants, it is very confusing so far, the summary shows the information that 23 interviews were carried out and in this section the authors describe that 18 women were invited to the study. It is necessary to clarify.

Data analysis

In this sentence is necessary to point the reference: Graneheim and Lundman method.

Still about the method

I would like to know a little more about who carried out the interviews, the setting period, expertise, room preparation, among other information that they deem important to show this relationship between researcher and research, as well as I would like to understand how internal and external validation of the study.

Did the authors use any instrument as a guide for writing the text?

Results

I think again that the question of the sample of 18 women and 23 interviews is very confusing, is it necessary to clarify how this process was, interviewing some women twice? How was this planned? I couldn't understand it clearly.

In the same way, I miss a better organization to talk about the categories built so that all the information doesn't get loose in the paragraph.

Subcategory 1.1 is unnamed

Discussion

The authors use in most of the text the concept of body perception and in the discussion they use the concept of self-image. I believe that it is more appropriate to use only one concept (body perception) in the entire text so as not to confuse the reader about which concept they are dealing with.

The authors bring some concepts: positive emotions and negative emotions. I would like to know what references were used to name emotions like that.

If the references do not bring it this way, I believe that a better option is to review the use of these concepts in the writing of the text.

I suggest this because in mental health, for example, there is not always a positive-good and negative-bad relationship, so when I read these excerpts I infer that this relationship was thought by the authors in this positive-good and negative-bad sense and that this can confuse the reader.

There are some very long paragraphs in this section, I suggest better dividing the information in the text to ensure a better “flow” of reading.

Table 1- I have doubts about the difference between diploma and Bachelor’s degree

Table 2 - table 2 I believe is not necessary, it is already described in the text of the results section.

Interesting would be a figure or even this table with data that explain how the categories were built, as was the reasoning of the authors.

Thanks again, success to the authors.

## **Reports v4**

## Reviewer 3

Body perception in pregnant women: a qualitative study

General Feedback

Overall, the theme discussed in the article is relevant and is within the scope of BMC Nursing. The purpose of the paper is good. To explore, qualitatively, body perception in pregnant women adds a lot of value to the field. I recognize that the authors have done a great job in correcting the manuscript in its previous version. However, some revisions are still necessary. I leave my comments below, specifying each section of the manuscript.

Abstract:

I could realize that the manuscript has now a methods sections in the abstract. I would appreciate if the authors include information about sample age in the abstract.

The authors could also include practical implications on the conclusion of the study in the abstract.

Background

-	I am in total agreement with Reviewer 2, concerning the need of a consistent conceptualization about your construct (Body Perception). I believe you may include the “Comment 4” of Reviewer 2 in your text. Besides that, you should cite some recognized authors during this conceptualization (e. g. Cash, Smolak, Thompson, Gardner, Brown).

-	Regarding comment 6 of Reviewer 22: On your new version of the text, you mentioned that: “Przybyła-Basista et al.’s study showed that body acceptance during pregnancy was one of the predictors of prenatal depression [8].”

In order to clarify that Body acceptance and prenatal depression have a negatively relationship in pregnant women, I believe that the correctly sentence should be: “… study showed that low body acceptance during pregnancy was one of the predictors of prenatal depression.”

-	I also agree with Reviewer 2 “In general, Introduction does not establish the background of the problem studied.” .

I believe you cold improve your background specially in two aspects:

1)	A better conceptualization about body image and its component “body perception” based on recognized authors.

2)	You may include evidence about previous studies that may considerer pregnant women’s body image in different cultures, so that you can compare to Iran culture.

Methods

Sampling:

-	I strongly suggest removing the sentence: “The completion of 5 interviews was postponed to another day on the participants’ preference.”. This makes no sense in the text and causes a lot of doubts for the reader.

Data collection:

-	I could understand that you include information about who carried out the interviews to attempt the Reviewer 1. But the status of “Ph.D. candidate” is temporary. I mean that, if someone read your publication article like 5 years later, you may not be a “Ph.D. candidate” any more. I suggest you to rewrite the sentence.

Results:

-	Please remove the sentence: “Due to the participants’ conditions and preferences, five interviews were conducted on two successive days.”

This information is unnecessary.

-	You mentioned a Table 2, but it is not available on the documents. If you remove the table 2, please, also remove the mention about it.

-	Please enumerate the three categories as the same way you did on Abstract.

Discussion.

Every study always has some limitations to point out. You have pointed only one limitation. Reviewer 2 have indicated some limitations you should include in your discussion (e.g. low sample size, lack of quantitative measures, and no fidelity checks within data analysis) I highly recommend reviewing the limitations of the study, including a full paragraph about it.

Conclusion

-	It is really important to NOT GENERALIZE your findings. Your study is a very small study (18 pregnant women) from a specific social context. So you must conclude your study taking in account the impossibility of generalization. Please rewrite the conclusion.

-	Practical implications should be better explored on conclusion.

Table 1

-	I suggest you to reordination the table columns, as follows: Age, GA; BMI; Educational level; Employment status; Monthly income (Rials).

Top of Form

Bottom of Form

Top of Form

Bottom of Form

## Reviewer 2

Dear editors and authors,

Thank you for the opportunity to review this interesting manuscript.

I believe the document is ready for publication.

Success to the authors

# **Reports v5**

## **Reviewer 3**

I acknowledge that the authors have made efforts to meet previous requests.

I believe the document is ready for publication.
